# The Labour Party and a State Medical Service

**Published:** 1919-05-03

**Authors:** 


### THE LABOUR PARTY AND A STATE MEDICAL SERVICE.

A committee appointed by the Labour Party to consider the Ministry of Health, in its relation to preventive and curative medical work, has reported in favour of a public medical service. The scheme advocated follows the lines made familiar to our readers by Sir Bertrand Dawson and Col. Maurice. Each local health area is to have a medical organisation run on the lines of the chapter of a skilled craft. Each chapter would have control of its professional affairs and, working with the local lay health committee, would be responsible for the health of the area served. Each chapter would have a principal medical officer under him, chief clinical medical officers supervising special departments such as those for mental disorders, sanitation, housing, maternity and child welfare, sanatoria, hospitals, pensions, domiciliary treatment, etc. The chapter would consist of general practitioners and specialists, with a hospital and consultative centre for headquarters and with laboratories for diagnosis and research. The public medical service would be free and open to all, irrespective of their financial and social position. The patient is to have free choice of a doctor, private practice is to be permitted, and no patient or doctor is to be forced to take part in the service against his wishes. The public service is to be a whole-time one, the scale of remuneration adequate to the services rendered, the hours of work are to be regulated, with holidays on the Civil Service scale and special study leave at the expense of the service; compensation for any disturbance of office and a reasonable pension on retirement are to be given. No practitioner is to have charge of more than 3,500 patients in urban districts, or 2,000 in rural districts. The cost of the public medical service is to be a charge upon communal funds, with substantial grants in aid and special help in large industrial areas where the hygienic conditions are bad and the rateable values low. It is recognised that there must be a transition period before the goal of a free medical service, staffed by whole-time salaried medical officers, is reached.

The scheme thus outlined is an attractive one, but the adherence of the medical profession to it depends upon details of remuneration, terms of service and organisation. The demand for a public whole-time medical service is now being voiced by many both without and within the profession. As we have said before, it behoves medical men to organise if they are to have the influence they ought to have in the settlement of this question of paramount importance to themselves and the community. A move in the right direction has been made by the Medical Parliamentary Committee. It has called for a conference of representatives of medical societies and organisations interested in health questions for May 2. Such a conference should lead to the formation of a body able to express the opinions of a large section of the medical profession. Its weakness lies in the fact that the general practitioner as such will be poorly represented upon it. Every small sectional interest will have its representative. Whence will come the representatives of the general practitioners? Apparently only from the British Medical Association. If that is the case the conference will be a lopsided one. One of the first duties of the conference will be to ensure the due representation of the general practitioner. The usefulness of the work it does will depend upon its success in this.

